# Case of General Paralysis

**Published:** 1849-04

**Authors:** S. L. Hollingsworth

**Affiliations:** Philadelphia


### Case of General Paralysis. By S. L. Hollingsworth, M. D., of Philadelphia.

J. P., aged 65 years, had been subject, during his youth, to convulsions, which were probably, from his account, epileptic. After their cessation, excepting an occasional attack of rheumatism, his health had been very good. In the early part of June, 1847, on attempting to mount a horse, he felt, for the first time, a slight weakness in his lower limbs. To this he paid but little attention, attributing it to rheumatism. It continued, however, to increase, though slowly, causing him to trip, when walking, and making his ascent to his room very slow and tiresome. His upper limbs soon afterwards were affected, so that, from being a hearty, strong, robust man, he became, in the course of two or three months, feeble in all his limbs, and tottering in his gait. His bowels, during this time, were costive, his head ached frequently, and his appetite was poor.

On the 1st of September, in passing through the garden to his house, he fell, and being unable to rise, had to lie upon the ground until some of his family came and assisted him to his room. On the 4th, the date of my first visit, I found him in bed, and obtained from him the above history of his case.

On examination, it appeared that voluntary motion of his upper and lower limbs was nearly gone, and that the only part of his body he could move with freedom, was his head, the muscles of the neck being unaffected, and continuing so during the whole period of his disease. He lay upon his back, and on desiring him to raise his arm, I found he did it with great difficulty. The motion was slow and tremulous. His intellectual faculties, senses, and power of sensation seemed unimpaired. He had no trouble in voiding his urine, and complained of no pain on pressure upon the vertebræ of the neck. His skin was dry, and his pulse weak and slow. Respiration and motion of heart natural.

His family informed me, that for many years past his habits had been rather intemperate.

Such was his condition at my first visit. From that time forward, his paralysis increased, until he was unable to help himself in the slightest degree, and it was necessary to wash, change, feed and lift him in and out of bed, precisely as if he were an infant. There came on, also, some loss of sensibility of the skin, but it was not very decided.

Great care had to be taken to prevent bed sores, by shifting small pillows under him, and by keeping him clean.

About the beginning of the next year, by which time he had become much emaciated, having lost at least 40 pounds, his appetite began to increase, and he thought he could move his limbs a little better. There was really some improvement, which continued until the month of April; he could assist himself considerably, and in June was able to leave his room, to which he has not since been confined. At present, he can walk without fatigue, three or four miles in a morning, and though not so strong as formerly, possesses a very fair share of strength in both his upper and lower extremities.

The treatment was little, and consisted in keeping his bowels free, for which purpose he took two compound rhubarb pills every second or third night, as he found necessary. I prescribed also the following: ℞. Potass. iod. ʒj, aquæ cinnam. ℥vi, ft. sol. S., a table spoonful thrice daily; but as its use during two months did not produce any sensible curative effect, it was discontinued. He refused to let me put a seton in his neck, as he feared its presence would prevent the motion of his head.

From the gradual increase in severity of the symptoms, I am inclined to think there was inflammation of the membranes of the spinal chord; and as respiration was unaffected, the seat of the disease must have been below the origin of the pneumogastric nerves. If there had been softening or ulceration of the spinal marrow, it is probable he would never have recovered. Other symptoms too, such as pain, spasms, &c., would have presented themselves. Whatever may have been the nature of the affection, the vis medicatrix naturæ must have the credit of his cure. I cannot help observing, that it would have been an excellent case for the powerful remedies of the homœopathists.

